# Speaking and thinking in Latin: some dead languages are alive and thriving in some very modern disorders

**DOI:** 10.1192/bjb.2025.20

**Published:** 2026-02

**Authors:** Jane Whittaker

**Affiliations:** Centre for the History of Science, Technology and Medicine, University of Manchester, Manchester, UK

**Keywords:** History of psychiatry, anorexia nervosa, arts psychiatry, feeding or eating disorders, philosophy

## Abstract

As psychiatrists, we are hopefully especially attuned to the power of language, especially the words we use when discussing sensations, thoughts and bodies. This article explores some of the heritage of medical language of today, drawing on classical Latin and how this interfaces with our day-to-day practice, with special reference to eating disorders.

Until the 1950s, you would have struggled to be accepted at medical school without some fluency in Latin. Only a century before, no gentleman’s education was complete without a grounding in the classic languages and literature, starting with Latin; and, if you were bright enough, you would be expected to learn Greek, and maybe Hebrew. Now, especially with English as the ubiquitous language of commerce, science and medicine, is there any point to being curious enough to spend time and effort on learning Latin? In this discussion, I want to propose that it most definitely is worthwhile, even as a former eating disorder psychiatrist who has worked with patients prsenting problems thought to be synonymous with modern life. Indeed, I would suggest that, without realising it, we are all chatting in Latin, albeit badly spoken, especially in medicine. It is mashed up with words taken from Norman French, a bit of Old German and a few recent arrivals from the Indian subcontinent. Anyone for a curry in the bungalow?

To most people, Latin is a dead language. It might have been of interest in the unlikely event that you wanted to converse with the administrative infrastructure of the Holy See in Rome before 2014, when it was the official language of Papal government.^1^ However, even their documentation is now in Italian. Beyond that, Latin might seem to have little role in modern cultural and scientific consciousness. However, take a closer look and you find that Latin was the language of science and medicine well into the 19th century. Linnaeus utilised Latin to frame his classification of living things, plants, animals and us, in the 18th century. Londa Schiebinger posed the question: ‘Why are mammals called mammals?’, a deceptively complex question that she answers with skill and scholarship; mammal is derived from the Latin ‘mamma’, meaning breast.^2^ The distinctive feature of mammals, that they suckle their young, is conveyed in Latin. *Homo sapiens* is Latin, ‘homo’ – ‘man’ or ‘human’ and ‘sapiens’ meaning wise – although use of the term ‘wise’ seems a bit off the mark given the state of the planet. We, as humans, give our name as a species to the Anthropocene, derived from Greek by the way – ‘anthropos’, meaning human and ‘cene’ from ‘kainos’, meaning new.

The language of modern medicine, including psychiatry, is full of Latin, from the gentleman physicians of the 17th century onwards. Medical care then came in many forms. Extrapolating from N.D. Jewson’s classic paper, one might argue that physicians catered to the landed classes. The apothecary probably took care of the servants.^3^ The local wise woman, perhaps doubling as the village midwife, provided care to everyone else. Her language of (usually) locally sourced herbal interventions would have been expressed in her local vernacular. I suspect her treatments, compared with those of the well-read physician, were no more efficacious, but probably less deadly. The gentleman physician’s interventions would have been grounded in humoral medicine, in Jewson’s argument offering a greater parity of intellectual interaction. This physician would probably have read Galen (129–216 CE), who was physician to Emperor Marcus Aurelius and author of one of the largest bodies of Greek literature extant, surviving arguably because his medical works were in use for centuries. Written in Greek but translated into Latin, including some sources via Arabic, and later into English and other modern vernacular languages, these works were being used as medical texts into the 18th century.^4^ Galen’s writings on psychological matters have been re-examined recently and make fascinating reading.^5^ Humoral conceptualisation of illness and health was the model for how diseases were framed. The same four humours – black bile, yellow bile, phlegm and blood – were balanced in health, and unbalanced in illness, whether that be what we might understand as physical or psychological. Ideally, the body, mind and soul existed in harmony with the environment. Interventions were largely founded around a recommended regime (from the Latin ‘regimen’) based on aiming for balance of the bodily humours, targeting diet, exercise and the quality of the air. A remedy (Latin, ‘remedium’) might have been suggested. Treatments ranged from bleeding, to blistering and a range of medications, sometimes harmless, at worst lethally toxic. Incidentally, ‘medicus’ is Latin for physician, but the term doctor is derived from ‘doceo’, meaning ‘I teach’, worth reflecting on in our current modern psychiatric practice? Our learned physician might have been able to converse with his patient in Latin and Greek, but it is likely that he was impressing his patient with his knowledge of classical languages and literature, much as the language of modern medicine continues to confuse, baffle and distract patients today.

## Old languages and modern disorders

We think of eating disorders as a modern phenomenon. However, the Greek and Latin roots of the term ‘anorexia nervosa’ are instantly recognisable. In 1991, Brenda Parry-Jones painstakingly worked through medical texts from the 15th century onwards, picking apart thematic terminology describing physicians’ categorisations of atypical eating and looking at Greek, Latin, early French and early English usage.^6^ Anorexia/anorexie/anorexy derived from the Greek negative prefix an- and -orexis, meaning desire, urge, appetite. Found in Latin texts as far back as Cicero, this still implies lack of appetite, even though the concept of appetite versus hunger versus food refusal in our modern conception of anorexia nervosa is complex. Boulime/bulimy/bulimia also have a basis in Greek, meaning ‘ox hunger’ and implying a great hunger inducing consumption of vast amounts of food. There is also mention of a condition called ‘bulmus’ in a Hebrew rabbinic text from about 1800 years ago: ‘If someone is seized with a manic hunger (bulmus), they may feed him even unclean foods until his eyes light up.’ (Mishnah Yoma 8:6). So, this concept has travelled from Greek, into Hebrew and through Latin and into middle French as ‘boulisme’.

Richard Morton (1637–1698) is often cited as the author of the first description of anorexia nervosa, using the term ‘nervous consumption’ in his ‘Phthisiologia’, his treatise on the different forms of consumption that he recognised.^7^ Clearly using modern diagnostic labels for his nervous consumption is anachronistic. However, for the purposes of this discussion, it is significant that Morton wrote his textbook on consumption and described his understanding of the different forms in Latin, using Latinised Greek, ‘phthisis’, for the title of his ‘Magnum Opus’. His publishers were responsible for the translation into English. They argued in their preface, just after Morton’s dedication, on an unnumbered page addressed ‘to the reader’, that they did this to avoid what could be, in their view, inferior translations:

‘THIS Book had remained concealed from Vulgar Eyes in the Learned Language in which it was writ by the Author, had we not been certainly informed that the Translation of it was intended and attempted by other Hands.’

We, as modern medics, assume that he meant tuberculosis, but to Morton consumption meant wasting, from the Latin ‘consumptus’, ‘consumed’, reflecting the observation that the sufferer was being eaten away from within. The root verb is consumo, consumere, to eat among other meanings. The identification of the tubercle bacillus was three centuries away, but Morton distinguished phthisis, with pulmonary symptoms, from the consumption of melancholia and nervous consumption. In his Phthisiologia, Morton described his patient, Miss Duke, as ‘a skeleton clad with skin’; she ultimately begged him to ‘leave the matter to nature’ and subsequently died.

A century later John Leake (1729–1792), in his text on consumption, discussed the treatment of ‘nervous consumption’. He wrote in English but referred to the physician’s need for the ‘skills of Medea’ to cure the various forms of consumption. This supports the idea that medicine was steeped in the culture of the classics.^a^ Medea was the enchantress who helped Jason and his Argonauts to steal the Golden Fleece. He may have been publishing in English, but classical stories were clearly in his writing. While some of us remember Jason and his adventures from Saturday afternoon Ray Harryhausen films, the Medea of Greek mythology looms large in Book 7 of the ‘Metamorphoses’ by Ovid.^8^ That epic poem is part of the foundational literature of Western Europe.

Book 8 of the same poem includes the story of Erysicthon, who wants to build a new, grander palace. He instructs his men to cut down trees, but they hesitate, knowing that the grove is sacred to Ceres, goddess of crops, farming and fertility. Erysichthon dismisses their concerns and, in an act of sacrilege, hacks down the most sacred tree, which bleeds as if it was a bull being sacrificed. As punishment, Ceres calls on one of the mountain spirits to inflict unending hunger upon Erysichthon, who in consequence can never be satisfied. Ovid uses a female personification of hunger. He writes as follows:

‘Her skin was so hard and fleshless; the entrails were visible through it; her shrunken bones protruded under her sagging loins: her belly was merely an empty space; her pendulous breasts appeared to be strung on nothing except the cage of her backbone; her leanness had swollen all of her joints; the rounds of her knees were bulbous: her ankles were grossly enlarged to a puffy excrescence.’

**Fig. 1:**
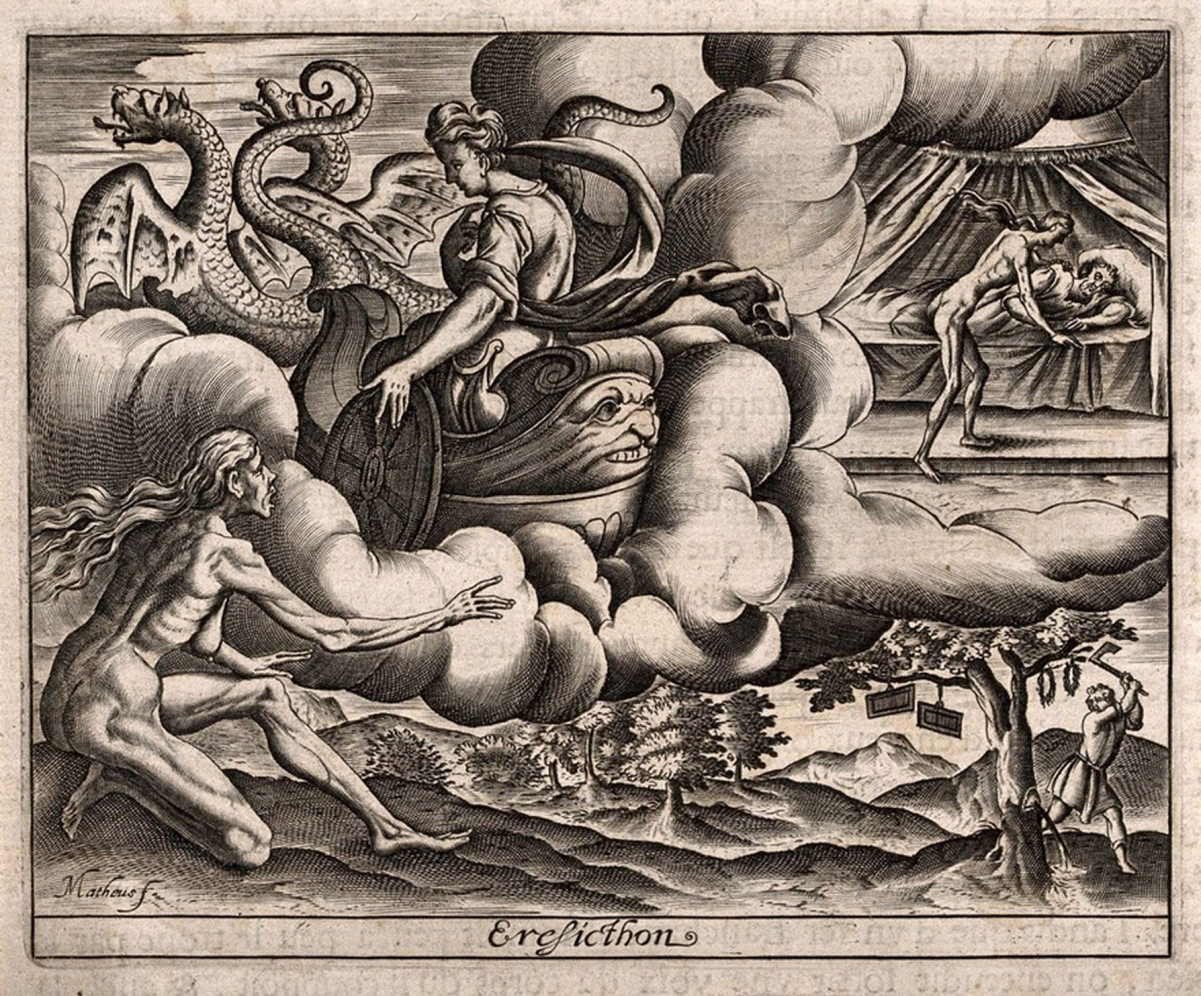
*Ceres punishes Erysichthon of Thessaly with perpetual hunger.* Engraving by J. Matheus, 1619. Wellcome Collection. Public Domain Mark. Source: Wellcome Collection (https://wellcomecollection.org/works/fdc89t3s).

Ovid, *Metamorphoses*, 8.800–10 (Fig. 1)

Those of us who have worked with teenagers and adults with severe eating disorders will instantly recognise this description of the severely emaciated female form, perhaps unaware that it is 2000 years old. Matheus’ evocative engraving tells the story of Erysichthon, including the figure of ‘Hunger’ – emaciated, bones visible and skin stretched over her sinewy musculature. Our patients often deny the subjective sensation of hunger, while others acknowledge an insatiable hunger yet are terrified at the thought of losing control and descending into an eating frenzy.

In the 19th century, William Gull (1816–1890) is typically credited with the first ‘modern’ description of what we now call anorexia nervosa.^9^ However, the notion that Gull invented the construction ‘anorexia nervosa’ is flawed, as Brenda Parry-Jones demonstrated. He toyed with the idea that anorexia nervosa was a disease of the stomach; words like dyspepsia, for example, are in his original report. I would suggest that his terminology was simply the latest in a long medical tradition of using neologisms rooted in other languages to try to describe, with precision, what a clinician was trying to make sense of when faced with a patient electively refusing to eat in the absence of obvious pathology.

## Foucault, Morton and for whom do we speak?

How thought is formulated into language is central to concepts that are being expressed and how those ideas are organised. Any psychiatrist who has listened to a patient with thought disorder knows this. Paul-Michel Foucault’s influence on the historical understanding of psychiatry is sometimes focused on his concept of the ‘great confinement’. The idea is that the Enlightenment ushered in a period of the primacy of reason. Those suffering from lack of reason (the mad, the criminal) had to be managed to be restored to reason. We might call it treatment and, thereby, recovery. Foucault would argue that we are forcing people back into conformity with what we consider to be ‘reason-able’. Foucault, however, also wrote at length about the way ideas are expressed, and how they evolve;^10^ he argued that language both liberated and constrained meaning, but that themes can be identified.^11^ In his Phthisiologia, Morton described his patient, Miss Duke, who begged that he let her be, a phrase familiar to anyone who has worked with patients with eating disorders, not least because ‘Let Me Be’ is the title of one of the most famous modern books on the subject.^12^

Our modern language of eating disorders is filled with the language of BMI, CBT and other statistical and therapy acronyms. How similar or distinct Morton’s ‘phthisis nervosa’ and our anorexia nervosa may be is open to debate, but both refer to young people who waste away to the exasperated bewilderment and distress of the people around them. Seventeenth- and eighteenth-century physicians were grounded in their classical education; they utilised Latin as a language that was both fluidly descriptive, highly precise and that conveyed both cultural context and scientific clarity, as they understood it. It facilitated international communication of ideas yet was also a language of an educated elite. It is cliche, but language is powerful, it includes and excludes. Professional language aids clarity, but the skill to share difficult concepts in difficult situations in clear language is also central to how we work. We might, with the condescension of the present looking back, pronounce some of the medical ideas of the past as mad, but we are still using their language even if we do not recognise it.

## Footnotes

a Accessed from the Special Collection of the John Rylands Library, University of Manchester, UK.
